# Dyadic concordance and associations of beliefs with intentions to learn carrier results from genomic sequencing

**DOI:** 10.1007/s10865-021-00222-0

**Published:** 2021-05-13

**Authors:** Chloe O. Huelsnitz, Erin Turbitt, Jennifer M. Taber, Katie L. Lewis, Leslie G. Biesecker, Barbara B. Biesecker, William M. P. Klein

**Affiliations:** 1 National Cancer Institute, Rockville, MD USA; 2 The University of Technology Sydney, Ultimo, NSW Australia; 3 Kent State University, Kent, USA; 4 National Human Genome Research Institute, Bethesda, MD USA; 5 Research Triangle Institute, International, Washington, DC USA

**Keywords:** Health beliefs, Genomic sequencing, Carrier results, Dyadic effects, Actor-partner interdependence model

## Abstract

Although romantic couple concordance has been demonstrated across a wide array of health *behaviors*, little research has examined dyadic concordance in health *beliefs*. This study examined the extent to which cohabitating romantic dyads’ attitudes and beliefs coincide (i.e., dyadic concordance) in addition to how well they predict intentions to learn genomic sequencing results. The actor-partner interdependence model was applied to cross-sectional data from 81 dyads in an exome sequencing study who were surveyed about their risk perceptions, worry, information avoidance, attitudes, and intentions toward learning carrier results. Information avoidance tendencies were positively correlated between partners, but there was low concordance on other beliefs. Individuals’ attitudes and information avoidance predicted their own intentions to learn results. Additionally, partners’ information avoidance tendencies predicted their partner’s intentions to learn results. Future research should explore mechanisms through which one’s partner’s information avoidance may affect one’s own intentions and behaviors.

## Introduction

Ample evidence has shown that romantic couples are concordant in health behaviors such as physical activity (Myers Virtue et al., 2015), smoking (Christakis & Fowler, 2008), and alcohol consumption (Torvik et al., 2013). However, fewer studies have examined dyadic concordance in health *beliefs* (e.g., perceived risk of developing a health outcome) or the extent to which partners’ health beliefs predict each other’s health behaviors and outcomes. One domain in which partners’ health beliefs may affect each other’s behavior is genomic sequencing—specifically pertaining to detection of carrier status for autosomal recessive conditions that could be passed on to a couple’s offspring. For couples who procreate, not only are their own carrier sequencing results valuable; their partner’s results are also valuable and important for understanding their descendants’ risk of having a child with a genetic condition.

From a clinical perspective, there is utility in understanding partner influence on intentions to learn carrier results. Social exchange processes—such as how and why partners influence each other’s health behaviour—often play minor roles in traditional theories and research on health decision-making, yet they offer a promising avenue for clinical interventions because partners can have potent effects on each other’s behavior (Rothman et al., 2020). In fact, couple-oriented interventions can be more efficacious than traditional psychosocial interventions at changing behavior (Martire et al., 2010). In the context of decisions regarding genomic sequencing, family members, including partners, may be particularly influential, as the sequencing results have implications for the family, rather than solely affecting the individual.

Research has shown that one’s own health beliefs—such as information avoidance (Taber et al., 2015b), attitudes and perceived social norms (Facio et al., 2013), and optimism (Taber et al., 2015a) play an important role in one’s own decisions to learn genomic carrier results. Many of these variables are considered standard in health behavior theories. However, few studies have examined the role of one’s romantic partner’s beliefs in the decision to learn carrier results. One study found that couples in which both members were more comfortable with the screening process, were highly knowledgeable about the process, and perceived fewer barriers, were more likely to participate in carrier screening than couples who reported low comfort, low knowledge, and more barriers (Henneman et al., 2001). Another study found that although worry about risk of a genetic condition in the family and intentions to share carrier results with family were correlated between partners, there were no associations of one’s partner’s attitudes, beliefs about the perceived value of results, or worry on one’s own intentions to share results with one’s family (Turbitt et al., 2018). The proposed analysis builds on the latter research by (a) incorporating additional constructs that have been shown to be associated with individuals’ own genome sequencing behavior [e.g., information avoidance (Taber et al., 2015b)] and b) assessing intentions to learn genome sequencing results.

## Methods

### Participants and recruitment

Individuals in the greater Bethesda, Maryland area and Washington, DC area were recruited to participate in a clinical exome sequencing study called ClinSeq® (Biesecker et al., 2009). Only participants in the additional cohort were financially compensated (the original cohort was not). They were informed that they would receive some medically useful results (e.g., blood chemistries and echocardiograms) and that they may have the opportunity to receive genomic sequencing results if they desired. An analysis of respondents from the first cohort indicated that many were motivated by altruism in addition to a desire to learn personalized genetic information (Facio et al., 2013). The original cohort (*n* = 1001 individuals, 68 individuals had spouses also enrolled in the study) was predominantly comprised of White, non-Hispanic/Latino individuals (Lewis et al., 2015). An additional cohort of individuals who self-identified as African, African American, or Afro-Caribbean were recruited (*n* = 467 individuals, 13 individuals had spouses also enrolled in the study) (Lewis et al., 2019). Participants in both cohorts were eligible if they were 45–65 years old at time of consent, had not smoked in the past year, and were not enrolled in another sequencing study that returned results. Recruitment to both cohorts was done using a variety of strategies including posting fliers in local businesses, staffing tables at community events, and word-of-mouth referrals by enrolled participants. See Biesecker et al. (2009) and Lewis et al. (2019) for additional recruitment information. Participants enrolled with the understanding that they may not learn sequencing results. This study was approved by the National Human Genome Research Institute Institutional Review Board. All participants provided written informed consent.

Participants were not intentionally recruited in dyads; couples were identified in the original cohort and additional cohort in one of three ways; (1) if they indicated on the baseline survey that their spouse was a ClinSeq® participant, which was verified by street address, (2) if participants had the same street address and last name as another participant, or (3) if they self-disclosed to a study staff member that their spouse was also in the study. Only dyads in which both members reported having the same number of biological sons and daughters were included in the current analyses (*n* = 162 individuals/81 dyads).^1^ See Table 1 for sample characteristics.

**Table 1:** Descriptive statistics, dyadic concordance, and bivariate correlations between predictors, outcome, and covariates

	Mean (SD) or proportion	ICC [95% CI]	1	2	3	4	5	6	7	8	9	10	11	12	13
1. Intentions (*N* = 159)	74.2% chose response of “7”	0.07 [− 0.16, 0.29]	1												
2. Risk (N = 159)	3.86 (1.95)	0.09 [− 0.13, 0.31]	− 0.11	1											
3. Worry (*N* = 162)	2.88 (1.50)	0.11 [− 0.11, 0.32]	− 0.19*	0.14	1										
4. Attitudes (*N* = 162)	6.66 (0.73)	0.05 [− 0.17, 0.26]	0.47**	− 0.14	0.04	1									
5. Information avoidance (*N* = 162)	2.03 (1.14)	0.22* [0.01, 0.41]	− 0.49**	0.09	0.06	− 0.45**	1								
6. Sex	50% Female	N/A	− 0.04	− 0.17*	− 0.07	0.01	0.00	1							
7. Age	60.58 (6.41)	0.85*** [0.77, 0.90]	0.00	− 0.00	0.03	0.00	− 0.03	− 0.12	1						
8. Education	90% College Degree or Higher	0.06 [− 0.17, 0.28]	0.03	0.12	− 0.06	− 0.14	− 0.01	− 0.16	0.15	1					
9. Household income	97% reported incomes above $100,000	N/A	− 0.01	− 0.10	− 0.12	− 0.03	− 0.09	0.00	0.23	0.32**	1				
10. Ethnicity	98% non-Hispanic/Latino	0.49*** [0.31, 0.64]	0.00	− 0.11	0.01	0.07	− 0.14	0.00	− 0.06	0.13	0.03	1			
11. Race	82% White	0.66*** [0.52, 0.77]	0.02	0.14	0.12	− 0.01	− 0.01	− 0.02	0.30**	0.22**	0.19*	0.05	1		
12. Ever had a genetic test	76% had never received a genetic test	0.27** [0.06, 0.46]	0.01	0.05	0.02	0.03	− 0.16*	0.12	0.01	0.05	0.10	− 0.09	0.22**	1	
13. Cohort	84% Original cohort	N/A	− 0.02	− 0.12	− 0.13	− 0.01	0.02	0.02	− 0.42**	− 0.24**	− 0.20*	− 0.07	− 0.92**	− 0.24**	1

*SD* standard deviation, *ICC* intraclass correlation coefficient, *CI* confidence interval

### Measures

The baseline survey included measures of social and behavioral constructs adopted from health behavior theories (e.g., the Health Belief Model, the Theory of Planned Behavior) and literature on common predictors of health information seeking were included in the baseline survey which was completed after informed consent but prior to receipt of any genetic testing results. The surveys were collected via mail, or during consent visits (verbally or on paper). The surveys completed by the two cohorts were slightly different; any differences in the outcome variable, predictor variables, and covariates are described below.

#### Outcome variable: intentions to learn carrier results

Intentions to learn carrier results were assessed with one item, which was preceded by the following prompt: “By participating in the ClinSeq® study and having your genome sequenced you could learn about a gene variant that does not affect your health, but may be important to the health of other relatives, such as your children.” The item was: I intend to learn such a result (1 = *definitely no* to 5 = *definitely yes*) (*M* = 6.51, *SD* = 1.20). The item was highly skewed (skewness = − 3.48), similar to analyses conducted only among participants in the original cohort (see for example, Ferrer et al., 2015). Square root transformation and log transformation did not reduce the skewness; thus, a median split was used to create a dichotomized version.

#### Predictor variables: information avoidance, risk perceptions, worry, and attitudes

Information avoidance was assessed as the average of six items (Howell et al., 2014)^2^ assessing preferences for learning information about one’s health (1 = *strongly disagree* to 7 = *strongly agree*) (Cronbach’s alpha = 0.71). A sample item is “I would rather not know everything about my health.” Higher scores reflect greater information avoidance. “Risk perceptions” were assessed with one item: “I feel like my relatives could be affected by a genetic condition that I have passed on” (1 = *strongly disagree* to 7 = *strongly agree*). Worry was assessed with one item: “How worried are you about the following outcomes? That your relatives could be affected with a genetic condition that you have passed on (1 = *not at all worried* to 7 = *extremely worried*).” Attitudes toward learning carrier results were assessed using a six-item scale (Facio et al., 2013; Michie et al., 2003). Participants indicated their response on 7-point Likert-type scales, in reference to carrier results to: “for me, learning such a result would be…” Scores ranged from 1, indicating negative attitudes (e.g., “*not a good thing*”, “*unimportant*”) to 7, indicating positive attitudes (e.g., “*a good thing*”, “*important*”). Cronbach’s alpha was 0.94.

Participant characteristics were collected and included as covariates: age, sex, race, ethnicity, education, household income, and whether participants had a previous genetic test result. Additionally, cohort membership was included as a covariate.

### Statistical analysis

Dyadic effects were examined using the actor-partner interdependence model (APIM) (Kenny et al., 2006). The APIM was used to simultaneously estimate the association of one’s own predictor variables on one’s own outcomes (i.e., actor effects) and the effects of one’s partner’s predictor variables on one’s own outcomes (i.e., partner effects). Data were analyzed using multilevel modeling with generalized least squares (GLS) estimation using the *nlme* package in R (Pinheiro et al., 2020). Actor effects tested were associations between all predictors (own risk perceptions, worry, attitudes, and information avoidance) and one’s own intentions to learn carrier results. Partner effects tested were associations between all predictors (one’s partner’s risk perceptions, worry, attitudes, and information avoidance) and one’s own intentions to learn carrier results. Covariances between each partner’s predictor and outcome variables were also modeled. Data were treated as indistinguishable, meaning that no systematic method was used to order the scores from the two dyad members (e.g., by sex or age).^3^

## Results

### Dyadic concordance

Dyadic concordance across the key study variables was assessed using intraclass correlation coefficients (ICC) (see Table 1). Information avoidance was positively correlated between partners (*ICC* = 0.22, *p* = 0.025); when individuals expressed higher tendencies to avoid health information, their partner did as well. Risk perceptions, worry, attitudes, and intentions to learn carrier results were uncorrelated between partners.

### APIM analysis: actor and partner effects

The results of the APIM models (with and without covariates) are shown in Table 2. People who were less worried that their relatives could be affected by a genetic condition that they have passed on reported greater intentions to receive carrier results (*b* = − 0.05, *p* = 0.023). However, this effect was not significant when covariates were included (*b* = − 0.04, *p* = 0.131). Individuals with more positive attitudes toward learning carrier results reported greater intentions to receive carrier results (*b* = 0.14, *p* = 0.012). Moreover, participants with greater tendencies to avoid information reported lower intentions to learn carrier results (*b* = − 0.12, *p* = 0.004). Risk perceptions were not significantly associated with one’s own intentions to learn carrier results.

**Table 2:** Results of APIM predicting actor intentions to learn carrier results

Predictor	Without covariates	With covariates
	Estimate (S.E.) [95% CI]	Estimate (S.E.) [95% CI]
Intercept	0.76*** (0.03) [0.71, 0.82]	1.01* (0.43) [0.16, 1.86]
Actor risk perceptions	− 0.00 (0.02) [− 0.03, 0.03]	− 0.00 (0.02) [− 0.04, 0.04]
Partner risk perceptions	0.01 (0.01) [− 0.02, 0.04]	0.00 (0.02) [− 0.04, 0.04]
Actor worry	− 0.05* (0.02) [− 0.09, − 0.01]	− 0.04 (0.03) [− 0.10, 0.01]
Partner worry	− 0.00 (0.02) [− 0.04, 0.04]	0.00 (0.03) [− 0.06, 0.06]
Actor attitudes	0.18*** (0.04) [0.09, 0.27]	0.14** (0.06) [0.03, 0.25]
Partner attitudes	− 0.07 (0.05) [− 0.16, 0.02]	− 0.06 (0.06) [− 0.18, 0.05]
Actor information avoidance	− 0.12*** (0.03) [− 0.18, − 0.07]	− 0.11** (0.04) [− 0.19, − 0.04]
Partner information avoidance	− 0.07** (0.03) [− 0.13, − 0.02]	− − 0.07^†^ (0.04) [− 0.14, 0.00]
**Model summary**		
Log likelihood	− 77.53	− 83.26
AIC	177.07	212.51
Pseudo R^2^	0.36	0.19

*AIC* akaike information criterion, *CI* confidence interval, *S.E.* standard error

^*^p < 0.05, **p < 0.01, ***p < 0.001, ^†^*p* < 0.10

Individuals whose partners reported greater tendencies to avoid information reported lower intentions to learn carrier results (*b* = − 0.07, *p* = 0.010). This effect became marginal when covariates were included (*b* = − 0.07, *p* = 0.067). Partners’ risk perceptions, worry, and attitudes were not significantly associated with actors’ intentions to learn carrier results.

## Discussion

The central contribution of this study is the finding that one’s partner’s information avoidance tendencies are associated with one’s own intentions to learn carrier results, even when accounting for one’s own information avoidance tendencies. Specifically, people reported lower intentions to learn carrier sequencing results if their partner had higher information avoidance. Arguably, learning carrier results can be beneficial, such that it could inform reproductive decisions of one’s descendants. If tendencies to avoid information serve as a barrier to obtaining this potentially useful information, then this may be a factor for genetic counselors to explore when meeting with patients. Another important finding was that there were no partner effects of risk perceptions, worry, or attitudes. This suggests that partner beliefs do not have equal effect on one’s own intentions and some beliefs appear to have greater effects than others.

An important next step for future research is to understand the mechanisms that explain how one’s partner’s information avoidance affects one’s own intentions to learn results. In order for one’s partners’ beliefs to affect one’s own behavior, it is likely that partners must communicate their beliefs to each other. Conversely, behaviors themselves are likely observable by one’s partner, which could explain the more frequently observed couple concordance among health behaviors (Christakis & Fowler, 2008; Myers Virtue et al., 2015; Torvik et al., 2013). In the current research, information avoidance was the only measure that is not specific to genome sequencing; rather, information avoidance was assessed with regards to health, more generally. Thus, it could be that partners have better awareness of each other’s overall interest in health information because there may be more opportunities for each partner to communicate and observe the other’s interest in health information in comparison with health beliefs that are context-specific. Future research can examine this question by assessing partners’ meta-beliefs, which refer to one partner’s beliefs about the other partner’s beliefs (e.g., “What are my partner’s attitudes about genome sequencing?”) and the extent to which those meta-beliefs are accurate.

### Limitations

There are several limitations in this research. One limitation is that carrier results were more relevant to participants’ grandchildren than to their children, as almost all participants were past reproductive age. Stronger partner effects may have been obtained if the results were more pertinent to reproductive decision making. A second limitation is that the carrier results were for health outcomes ranging from low to high severity and probability; the findings may be stronger if limited to more impactful genomic sequencing results. For instance, it is unknown to what extent findings would generalize to contexts where individuals can learn about genetic risk for severe diseases, such as genetic testing for high-penetrance familial risk such as *BRCA1/2* genetic testing for hereditary breast and ovarian cancer. A third limitation is that a single item was used to assess two of the predictors (risk perceptions and worry) and intentions, which could have made it difficult to obtain reliable effects for these predictors. This strategy is typical in large scale, nationally representative studies and one- and two-item measures of variables such as self-affirmation have shown predictive validity in other studies (e.g., Taber et al., 2015b).

A final limitation is selection bias. People who are willing to participate in studies like ClinSeq® are likely to be different from people who do not participate, such that they may be more interested in receiving information about their health. Overall tendencies to avoid information were low (the mean was 2.03 and 2 SDs above and below the mean = 0.89–3.17, which are below the midpoint of 4) and intentions to learn results were skewed and quite high, which makes sense given that the sample included people who were interested in receiving genomic information. We expect that stronger associations would be observed for people who are higher in information avoidance. These findings are likely to generalize to other individuals who undergo genomic sequencing and are presumably interested in learning genomic sequencing results. Additionally, dyadic studies are often prone to selection bias; for instance, people who participate in studies with their romantic partner are less likely to experience breakup than people who participate solo (Park et al., 2020). The findings from the current research will be most generalizable to couples who are interested in participating in genome sequencing together.”

### Clinical implications

Although some interventions and public health programs are beginning to incorporate close others as agents of influence, it is important to understand how social exchange processes function before implementing large-scale programs. For instance, pilot research studies of population-based carrier screening are currently being conducted in the Netherlands and Australia (Schuurmans et al., 2019; “What is Mackenzie’s Mission?”, 2019). In these programs, both members of the couple need to participate in order to learn their carrier results; thus, if one partner declines to participate, the couple is ineligible. The results of the current research have implications for these types of screening programs; people whose partners’ have higher information avoidance may be less likely to participate.

## Footnotes

1 64 individuals (32 dyads) were excluded who a) reported that either they or their partner do not have children (*n* = 62) or b) reported a different number of biological sons and/or biological daughters (*n* = 2).

2 The original cohort completed eight items assessing information avoidance, whereas the additional cohort completed six items. Only the common items were included in these analyses.

3 As the dyads included heterosexual and a homosexual couple, data were treated as indistinguishable.
